# Factors Affecting Axillary Lymph Node Involvement Based on Permanent Section Evaluation of the Excised Sentinel Lymph Nodes in Early-Stage Breast Cancer Patients: A Single-Center Retrospective Study

**DOI:** 10.3390/medicina62010213

**Published:** 2026-01-20

**Authors:** Hakan Baysal, Tunc Eren, Kubra Kargici, Ozge Kapar, Begumhan Baysal, Orhan Alimoglu

**Affiliations:** 1 Department of General Surgery, Goztepe Prof. Dr. Suleyman Yalcin City Hospital, Faculty of Medicine, Istanbul Medeniyet University, 34722 Istanbul, Turkey; drtunceren@gmail.com (T.E.); kubrakargici1@gmail.com (K.K.); orhan.alimoglu@medeniyet.edu.tr (O.A.); 2 Department of Pathology, Goztepe Prof. Dr. Suleyman Yalcin City Hospital, Faculty of Medicine, Istanbul Medeniyet University, 34722 Istanbul, Turkey; m.ozgekapar@gmail.com; 3 Department of Radiology, Goztepe Prof. Dr. Suleyman Yalcin City Hospital, Faculty of Medicine, Istanbul Medeniyet University, 34722 Istanbul, Turkey; baysalbegumhan@yahoo.com

**Keywords:** breast neoplasms, sentinel lymph node, lymphatic metastasis, pathology, general surgery

## Abstract

*Background and Objectives:* Sentinel lymph node (LN) biopsy (SLNB) remains to be the standard approach for surgical axillary staging of breast cancer (BC) patients. The aim of this study was to investigate the factors that affect axillary LN involvement in early BC patients. *Materials and Methods:* Clinically node negative early stage (cT1-2N0) BC patients having undergone breast conserving surgery (BCS) between February 2021 and January 2024 were included. During axillary exploration of all cases, sentinel LNs were excised and reserved for permanent section pathological examination (PS) only. Historical records of patients including clinicopathological features, surgical outcomes as well as pathological results were recorded and analyzed retrospectively. *p* < 0.05 indicated statistically significant results. *Results:* The study group consisted of 150 women with cT1-2N0 BC having undergone BCS with a median age of 59 (range: 25–81) years. According to the PS results of the sentinel LNs, the need for reoperation to complete axillary lymph node dissection was present in three (2%) patients. Tumors of the Luminal B subtype were significantly associated with increased sentinel LN positivity (*p* = 0.014). The risk of sentinel LN metastasis was found to be 5.2 times greater in patients with a Ki-67 ≥ %14 [OR: 5.224 (%95 CI:1.73–15.82, *p* = 0.003)] and the Ki-67 proliferation index was determined as an independent risk factor. *Conclusions:* In early-stage BC patients, PS of the excised sentinel LN offers sufficient axillary LN staging. On the other hand, a more careful clinical assessment is necessary for early BC patients harboring tumors with an elevated Ki-67 index and/or tumors of the Luminal B subtype.

## 1. Introduction

Breast cancer (BC) comprises 11.7% of all newly detected cancers and more than 2.2 million new BC cases continue to be diagnosed annually [1]. Screening programs and growing awareness of BC have led to increased rates of early diagnosis, resulting in a decreased incidence of axillary lymph node (LN) metastasis [2].

The presence of axillary LN metastasis is one of the most crucial prognostic factors for early-stage BC. The evolution of axillary surgical staging from axillary LN dissection (ALND) to sentinel LN biopsy (SLNB) has provided the benefit of diminished surgical morbidity without compromising oncological safety. Currently, SLNB has become the standard procedure of choice for axillary staging. In several clinical studies, it was shown that there was no significant difference in terms of both survival and regional LN recurrence among patient groups having undergone either SLNB, or ALND [3,4]. Patients with clinical tumor classifications of cT1-2 along with axillary node negative (cT1-2N0) BC are candidates for the omission of ALND when upfront surgery is determined to be the primary treatment [5]. As results of these clinical studies, ALND rates have significantly decreased worldwide. Despite being the routine method for the evaluation of sentinel LN intraoperatively, frozen section pathological examination (FS) is an underpowered diagnostic procedure yielding lower accuracy when compared to the permanent section pathological examination (PS) [6]. The reported false negativity rates of intraoperative FS range between 25% and 42% [7]. Additionally, intraoperative FS is associated with risks of permanent tissue loss of the axillary specimen for PS, extended operative duration, and increased medical costs.

In the present study, our aim was to investigate the factors that might affect sentinel LN positivity in surgically treated early-stage BC patients at PS.

## 2. Materials and Methods

The present study was conducted at the Department of General Surgery of our institution between February 2021 and January 2024. Ethics committee approval was obtained from the institutional review board (IRB) for the study [IRB number: 2022/0641], and signed informed consent forms were obtained from all patients. The present study was performed in accordance with the ethical standards laid down in the Declaration of Helsinki.

Historical records of the patients including demographics, clinicopathological features at preoperative assessments, treatment methods, and surgical outcomes as well as pathological examination results were retrospectively evaluated. In addition to their clinicopathological findings, patients were evaluated with use of mammography (MG), breast ultrasonography (US), and contrast enhanced breast magnetic resonance imaging (MRI).

Patients between ages of 18 to 90 years with the diagnosis of cT1-2N0 invasive BC having undergone breast conserving surgery (BCS) were included. Tumor size, location, and multifocality were assessed via MRI whereas US was used in patients for which MRI could not be performed. Patients with normal axillary imaging results were included, while fine needle aspiration biopsy (FNAB) was performed in patients with abnormal axillary LN findings. Patients with malignant FNAB results were marked under US guidance with the implantation of a gold marker within the metastatic LN for a future targeted axillary dissection and were excluded from the study. Patients eligible for BCS followed by radiotherapy (RT) were included, whereas patients requesting a mastectomy and patients who harbored any distant metastasis were excluded. A reoperation for completion ALND was recommended to and planned for patients who possessed at least three metastatic sentinel LNs. Furthermore, male patients, patients having undergone neoadjuvant therapy, and patients who harbored any concomitant malignancies were excluded from the study.

Intraoperative SLNB was carried out via the single-agent mapping tracer method. After anesthesia induction, 5 milliliters of 1% isosulphan blue dye was injected into the subareolar space. At axillary exploration, the blue-dyed axillary LNs were excised and reserved for PS only without requesting an intraoperative FS.

After the number, size, and gross appearance of the formalin fixed sentinel LNs were recorded, each intact sentinel LN was submitted for histopathological examination and paraffin-embedded sections were cut at multiple levels. Initial evaluation was performed on hematoxylin and eosin (H&E) stained sections. In cases where no metastasis was identified on routine H&E examination, additional step sections were obtained. Immunohistochemistry (IHC) for pancytokeratin (AE1/AE3) was performed with an automatic staining system (Leica Bond-III Fully Automated IHC/ISH staining system^®^, Deer Park, IL, USA) to detect occult metastases, including micrometastases and isolated tumor cells (ITCs), when indicated. Metastatic deposits were classified according to the 8th edition of the American Joint Committee on Cancer (AJCC) staging manual as macrometastasis (>2 mm), micrometastasis (>0.2 mm and ≤2 mm), or ITCs (≤0.2 mm or single cells). The presence or absence of extranodal extension was also documented.

Primary tumors were classified into four biologic subtypes according to their estrogen receptor (ER), progesterone receptor (PR), human epidermal growth factor receptor 2 (HER2), and antigen Kiel 67 (Ki-67) proliferation index status. ‘Luminal A’ tumors were defined as the presence of ER and/or PR, the absence of HER2, and a Ki-67 < 14%. ‘Luminal B HER2 negative’ tumors were defined as ER and/or PR positive, and HER2 negative that showed a Ki-67 ≥ 14%, whereas ‘Luminal B HER2 positive’ tumors were indicated by the presence of ER and/or PR in addition to an amplified HER2, along with any Ki-67 status. The ‘HER2 overexpression’ subtype tumors were defined as ER and PR negative along with an amplified HER2, while ‘triple negative’ tumors were indicated by the absence of ER, PR, and HER2.

Statistical analysis was performed using the Number Cruncher Statistical System^®^ (NCSS 2020 Statistical Software version 20.0.8, Kaysville, UT, USA). Additional to standard descriptive statistical methods (mean, standard deviation, median, minimum, maximum, frequency, ratio), the Shapiro–Wilk test, and box plot graphics were used for the evaluation of the normal distribution of variables. Student’s *t*-test was used to compare parameters between two quantitative groups with normal distribution whereas the Mann–Whitney *U* test was used for the comparison of the groups with any maldistribution of parameters. Pearson’s chi-squared, Fisher’s exact, and Fisher–Freeman–Halton tests were used for the comparisons of qualitative data. Diagnostic scans including sensitivity, specificity, positive predictive value (PPV), negative predictive value (NPV), and receiver operating characteristics (ROC) curve analysis were used in order to determine cutoff values for the parameters. Variables affecting LN positivity (according to the final histopathology results) that were found to be significant or close to significant by univariate assessments were included in the multivariate assessment which was carried out via logistic regression analysis. The results were evaluated as odds ratios (OR) with a 95% confidence interval (CI) and a *p* < 0.05 value was accepted as statistically significant.

## 3. Results

The study group consisted of surgically treated 150 women, all of whom were cT1-2N0 BC patients with a median age of 59 (range: 25–81) years; 53 (35.3%) patients were premenopausal and 97 (64.7%) were postmenopausal. According to the pathological examinations, the mean tumor size was detected to be 19.10 ± 9.29 (median: 17, range: 2–49) mm. Patients’ clinicopathological characteristics are summarized Table 1.

According to the final pathology results of the PS of excised sentinel LNs, significant difference was detected in terms of the Ki-67 proliferation index of the patients as a Ki-67 ≥ 14% was detected to be associated with a significantly higher sentinel LN positivity (*p* = 0.002). The PS of excised sentinel LNs also revealed that the number of Grade 1 tumors was significantly higher in the sentinel LN negative cases whereas Grade 3 tumors were associated with significantly increased rates of sentinel LN involvement (*p* = 0.012). Additionally, Luminal A tumors were more frequent in the sentinel LN negative group while the number of Luminal B tumors were significantly higher in the sentinel LN positive group (*p* = 0.014). On the other hand, variables including menopausal status, tumor classification, histological classification, ER, PR, and HER2 status were not found to affect sentinel LN positivity (*p* > 0.05) (Table 1).

The PS of excised sentinel LNs revealed macrometastasis in 23 (15.3%) patients and micrometastasis in 10 (6.7%) patients while the presence of ITCs was detected in 3 (2.0%) patients. At axillary exploration, the excised total LN count as well as the blue dye-stained LN count were higher in the sentinel LN positive group (*p* = 0.038 and *p* = 0.032, respectively). Three (2.0%) patients were detected to harbor three metastatic sentinel LNs at final pathology results. One (0.7%) of these patients accepted a reoperation and ALND was performed resulting with four metastatic LNs among 16 harvested axillary LNs at final pathological evaluations. The remaining two (1.3%) patients preferred a combined adjuvant therapy including chemotherapy, hormone therapy, and breast and axillary RT. In addition to surgical and pathological outcomes, mortality and special pathological findings are also summarized in Table 2.

**Figure 1:**
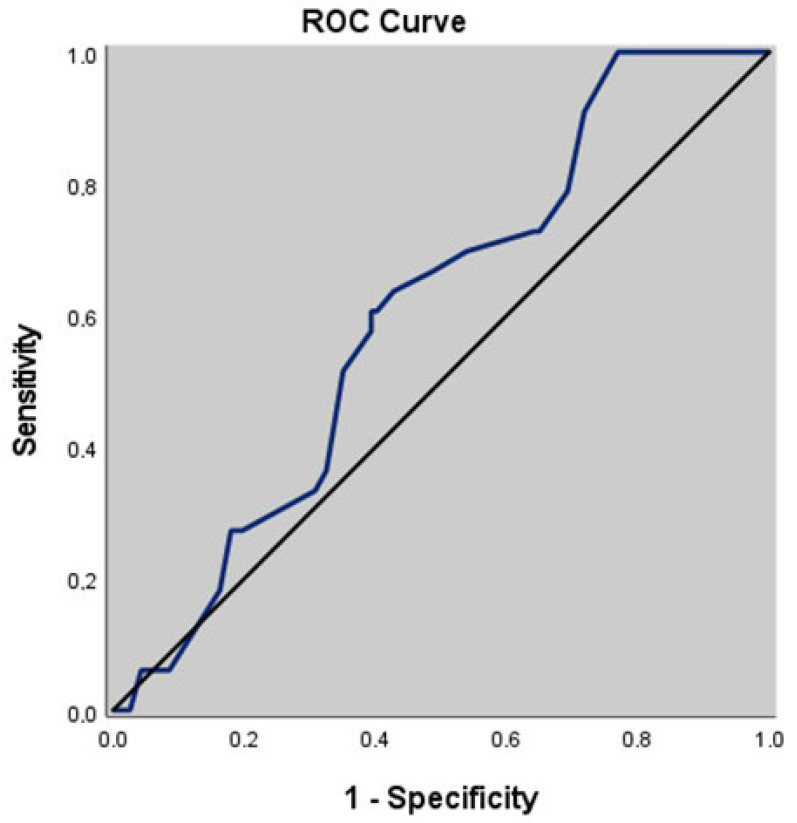
The receiver operating characteristics (ROC) curve for the relation of tumor size and sentinel lymph node metastasis.

When sentinel LN metastasis was taken into account, the cutoff tumor size was calculated as 19 mm, or above. Tumor size ≥ 19 mm was associated with a sensitivity of 57.58%, a specificity of 59.83%, a PPV of 28.79%, and an NPV of 83.33% for sentinel LN positivity. The area under the ROC curve was calculated as 60.3% (95% CI: 0.520–0.682) (Table 3, Figure 1). Although tumor size was not found to reach statistical significance related to sentinel LN positivity, tumor size ≥ 19 mm was considered to be associated with an approximately two-times greater risk of sentinel LN involvement [OR: 2.021 (%95 CI: 0.924–4.423, *p* = 0.076)].

**Figure 2:**
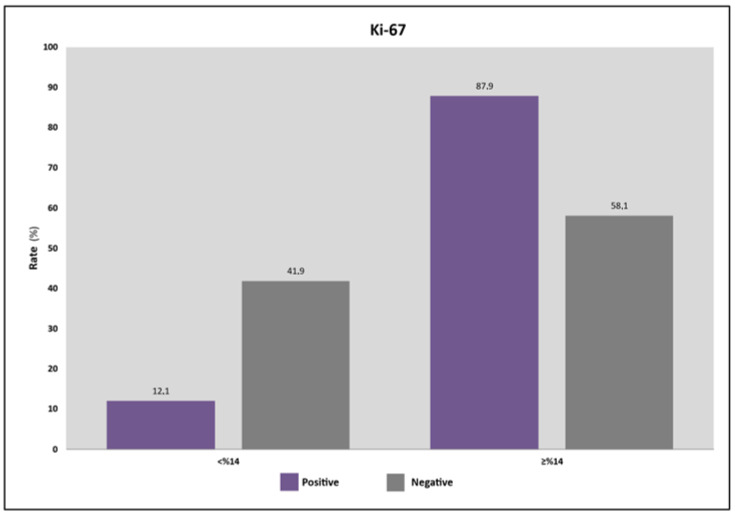
The association of antigen Kiel 67 (Ki-67) levels and sentinel lymph node (LN) metastasis. Ki-67 ≥ 14% was associated with a significantly higher sentinel LN positivity (*p* = 0.002).

Enter Logistic regression was used for the multivariate analysis of tumoral features that affect sentinel LN positivity. It was detected that the model was significant (F = 11.371; *p* < 0.01) and its demonstrative quotient was found to be 78%. Histological grade and tumor size were not found to be statistically significant according to the multivariate analysis. The risk of sentinel LN positivity was found to be 5.2 times greater in patients with a Ki-67 index of %14 or above [OR: 5.224 (%95 CI: 1.73–15.82, *p* = 0.003)], and therefore, a Ki-67 ≥ 14% was found to be an independent risk factor for sentinel LN metastasis (Table 4, Figure 2).

## 4. Discussion

As results of recent notable studies reporting the oncological safety of the omission of ALND in patients with a limited axillary LN burden, it would be fair to state that ALND rates have remarkably declined in the current practice of BC surgery. Accordingly, the requirement of FS for intraoperative sentinel LN evaluation has also decreased. The aim of the present study was to determine important risk factors to be taken into account that may increase sentinel LN positivity at PS in our refined study group consisting of clinically and radiologically node-negative early-stage BC patients having undergone BCS.

The evaluation of the axillary LN status is of utmost importance for cancer staging, decision making in adjuvant therapy modalities, and predicting the prognosis in BC patients [8]. Among developing surgical management strategies, the requirement of ALND has been the most questioned and studied issue.

The revolutionary ACOSOG Z0011 study has paved the way for the application of less radical procedures in the surgical treatment of BC, as it was concluded in this study that in eligible patients matching the study criteria, 10-year overall survival (OS) after SLNB was not impaired when compared to the patient group having undergone ALND [9]. It was also confirmed in the AMAROS study that the choice of axillary therapy in terms of either ALND or axillary RT did not affect survival in patients with positive sentinel LNs [10]. Although the omission of ALND lead to a tendency in increased axillary recurrence in cN0 BC patients, this slightly elevated local recurrence rate was not resultant with either any higher distant metastasis rates, or any survival disadvantage [9,10,11]. Currently, SLNB has replaced ALND for axillary staging in cN0 BC patients.

Recently, ALND is being infrequently performed even in select cases with metastatic sentinel LNs. Undoubtedly, the best way to define the patients who will benefit from ALND is the intraoperative FS of the excised sentinel LNs. However, accurate decision making is not always possible with FS as this method also has its own disadvantages. When compared to PS, FS detects morphologically smaller tissue sample sections, is harder to interpret, and the utility of IHC is limited. Intraoperative FS may be associated with permanent tissue loss of the axillary specimen which may lead to alterations in final pathology results [12]. Due to the variations in FS protocols, a wide range of false negativity, between 6% and 43%, has been reported in multiple studies [7,13,14,15]. In addition, FS extends the operative time under general anesthesia, requires the dedicated work of an experienced pathologist, increases medical costs, and is a time-consuming method [16]. Despite these disadvantages, studies questioning the convenience of the omission of ALND have advocated the routine implementation of FS [14,17,18].

Previous studies on FS and PS have investigated the preventive efficiency of these methods from the requirement of a reoperation, in terms of a completion ALND, in early-stage BC patients. The preventive effect of FS, which was reported to be 12% in a previous study conducted in 2018, was found to be 0% in a recent study published in 2022 [17,19]. This marked decline may be due to increased rates of neoadjuvant therapy practices being applied to early-stage BC patients with an increasing trend. In another study, a reoperative requirement for a completion ALND was reported to be 1.9% in the subgroup for which routine PS was utilized [16]. In our study, three (2%) patients were detected to require a reoperation for a completion ALND. One (0.7%) of these patients underwent a reoperation and ALND was performed, resulting in a final pathology result of four metastatic LNs among 16 harvested axillary LNs. The other two (1.3%) patients opted for a combined adjuvant therapy including chemotherapy, hormone therapy, breast and axillary RT, along with close follow-up.

In one of the initial and verified models concerning sentinel LN metastasis, eight clinicopathological variables were determined including age, tumor size, tumor location, lymphovascular invasion (LVI), multifocality, and the ER and PR states [20]. Multiple studies have also been conducted investigating other variables in addition to these given parameters. In a recent study, the authors reported that Nottingham Histological Grade (the combination of tumoral features including tubule formation, nuclear pleomorphism, and mitotic activity) and LVI were predictive factors for sentinel LN metastasis [13]. In another study, it was reported that neither age nor ER and HER2 states affected the axillary LN burden [21]. In studies investigating more than three metastatic sentinel LNs, larger tumor size and higher grade were found to be risk factors for LN involvement [22,23] whereas one study could not depict the effects of tumor biology (ER, PR, and HER2 states) on axillary LNs [24]. In their study of 436 patients, Aydin et al. reported that a higher grade and LVI were risk factors for sentinel LN metastasis and they found that the Luminal B subgroup of tumors imposed a higher risk [25]. Sentinel LN metastasis risk was detected to be higher in patients with higher tumor grade, absence of ER, presence of LVI, and larger tumor size in another recent study [26]. On the other hand, in their study of 147 patients, Salman et al. could not demonstrate an association of sentinel LN metastasis with age, tumor size, histological grade, tumor histology, Ki-67 index, ER, PR or HER2 states [27]. It is obvious that the aforementioned studies investigating different variables affecting sentinel LN involvement present heterogeneous results. Logistic regression analysis of our study revealed that histological grade had a significant impact on LN positivity as Grade 3 tumors were associated with significantly higher rates of sentinel LN metastasis.

In the present study, menopausal state, tumor size, tumor classification, histopathological features, and the ER, PR and HER2 states were not found to significantly affect sentinel LN positivity in early-stage BC patients. Although the Luminal B subgroup was associated with significantly higher sentinel LN metastasis, it is noteworthy that HER2 positive tumors, which constitute an aggressive subtype, were not associated with increased LN involvement. This issue may be explained by our neoadjuvant treatment preference for HER2 positive tumors that are classified as cT1c or a larger T stage. We calculated the cutoff for tumor size associated with sentinel LN positivity to be 19 mm. Although tumor size could not be determined to significantly affect LN involvement, tumors ≥ 19 mm were found to impose approximately two times higher risk for sentinel LN metastasis [OR: 2.021 (%95 CI: 0.924–4.423, *p* = 0.076)]. Moreover, it was found in our study that the risk of sentinel LN positivity was approximately five times greater in patients with a Ki-67 index of 14% or above [OR: 5.224 (%95 CI: 1.73–15.82, *p* = 0.003)] which demonstrates that the Ki-67 ≥ 14% value is an independent risk factor for sentinel LN metastasis.

This study has a number of limitations such as its retrospective nature and results obtained from a single surgical center. Since neoadjuvant therapy was preferred for patients with HER2 positive or triple negative tumors that are clinically classified as T1c or larger, the number of patients harboring these tumor types may have inevitably been altered. Nevertheless, we believe that the present study offers useful information regarding intraoperative sentinel LN management in clinically and radiologically node-negative early-stage BC patients. The requirement for reoperation was markedly low in our study group of patients in whom FS was omitted. Therefore, PS was considered as safe and useful for the axillary staging of early-stage breast cancer patients.

## 5. Conclusions

While we witness that axillary surgery is becoming less commonly practiced at the present time, we demonstrated in our study that in clinically and radiologically node-negative early-stage (cT1-2N0) BC patients, the sole use of PS was found to offer satisfactory results along with very low rates of the need for a reoperation in terms of a completion ALND.

On the other hand, we advise that surgeons should be more careful with their preoperative assessments of early BC patients diagnosed with tumors with an elevated Ki-67 proliferation index and/or tumors of the Luminal B subtype as such cases possess higher risk factors for sentinel LN positivity. Thus, it would be right to emphasize herein that a Ki-67 ≥ 14% was found to be an independent risk factor for sentinel LN metastasis in cT1-2N0 BC patients.

## Figures and Tables

**Table 1:** Comparison of patient and tumoral characteristics according to the permanent section pathological examination results of the excised sentinel lymph nodes.

Characteristics			*N = 150*	Sentinel Lymph Node Final Pathology Result		*p*
				Positive *(n = 33)*	Negative *(n = 117)*	
**Menopausal state**	Premenopausal	*n (%)*	53 (35.3)	14 (42.4)	39 (33.3)	^a^ 0.355
	Postmenopausal	*n (%)*	97 (64.7)	19 (57.6)	78 (66.7)	
**Breast side**	Right	*n (%)*	68 (45.3)	11 (33.3)	57 (48.7)	^a^ 0.117
	Left	*n (%)*	82 (54.7)	22 (66.7)	60 (51.3)	
**Tumor size** *(millimeters)*		*Mean ± SD*	19.10 ± 9.29	21.27 ± 8.04	18.47 ± 9.48	^b^ 0.130
		*Median (min-max)*	17 (2–49)	22 (11–40)	16 (2–49)	
**Tumor** **classification**	T1a	*n (%)*	5 (3.3)	1 (3.0)	4 (3.4)	^c^ 0.058
	T1b	*n (%)*	24 (16.0)	1 (3.0)	23 (19.7)	
	T1c	*n (%)*	56 (37.3)	12 (36.4)	44 (37.6)	
	T2	*n (%)*	65 (43.3)	19 (57.6)	46 (39.3)	
**Histological** **classification**	Invasive ductal	*n (%)*	107 (71.3)	23 (69.7)	84 (71.8)	^c^ 0.590
	Invasive lobular	*n (%)*	13 (8.7)	4 (12.1)	9 (7.7)	
	Mucinous	*n (%)*	3 (2.0)	0 (0.0)	3 (2.6)	
	Papillary	*n (%)*	19 (12.7)	6 (18.2)	13 (11.1)	
	Tubular	*n (%)*	3 (2.0)	0 (0.0)	3 (2.6)	
	Other	*n (%)*	5 (3.3)	0 (0.0)	5 (4.3)	
**Estrogen** **receptor**	Negative	*n (%)*	12 (8.0)	3 (9.1)	9 (7.7)	^d^ 0.727
	Positive	*n (%)*	138 (92.0)	30 (90.9)	108 (92.3)	
**Progesterone** **receptor**	Negative	*n (%)*	21 (14.0)	4 (12.1)	17 (14.5)	^d^ 1.000
	Positive	*n (%)*	129 (86.0)	29 (87.9)	100 (85.5)	
**HER2**	Negative	*n (%)*	136 (90.7)	27 (81.8)	109 (93.2)	^d^ 0.082
	Positive	*n (%)*	14 (9.3)	6 (18.2)	8 (6.8)	
**Ki-67**	<14%	*n (%)*	53 (35.3)	4 (12.1)	49 (41.9)	^a^ 0.002 **
	≥14%	*n (%)*	97 (64.7)	29 (87.9)	68 (58.1)	
**Histological** **tumor grade**	Grade 1	*n (%)*	36 (24.0)	2 (6.1)	34 (29.1)	^a^ 0.012 *
	Grade 2	*n (%)*	75 (50.0)	18 (54.5)	57 (48.7)	
	Grade 3	*n (%)*	39 (26.0)	13 (39.4)	26 (22.2)	
**Biologic tumor** **classification**	Luminal A	*n (%)*	52 (34.7)	5 (15.2)	47 (40.2)	^c^ 0.014 *
	Luminal B	*n (%)*	86 (57.3)	25 (75.8)	61 (52.1)	
	HER2 overexpression	*n (%)*	4 (2.7)	2 (6.1)	2 (1.7)	
	Triple negative	*n (%)*	8 (5.3)	1 (3.0)	7 (6.0)	

^a^ Pearson’s chi-squared test, ^b^ Student’s *t*-test, ^c^ Fisher–Freeman–Halton test, ^d^ Fisher’s exact test, N: Total number of patients, n: number of patients, ** *p* < 0.01, * *p* < 0.05, Mean ± SD: Mean ± standard deviation, Median (min-max): Median (minimum–maximum), HER2: Human epidermal growth factor receptor 2, Ki-67: Antigen Kiel 67.

**Table 2:** Comparison of surgical and histopathological characteristics according to the permanent section pathological examination results of the excised sentinel lymph nodes.

Characteristics			*N = 150*	Sentinel Lymph Node Final Pathology Result		*p*
				Positive *(n = 33)*	Negative *(n = 117)*	
**Lymph node** **positivity**	Isolated tumor cells	*n (%)*	3 (2.0)	0 (0.0)	3 (2.6)	N/A
	Micrometastasis	*n (%)*	10 (6.7)	10 (30.3)	0 (0.0)	
	Macrometastasis	*n (%)*	23 (15.3)	23 (69.7)	0 (0.0)	
**Metastatic** **lymph nodes**	*n = 33*	*Mean ± SD*	1.27 ± 0.62	1.27 ± 0.62	0 (0.0)	N/A
		*Median (min-max)*	1 (1–3)	1 (1–3)	0 (0.0)	
	1 lymph node	*n (%)*	27 (81.8)	27 (81.8)	0 (0.0)	N/A
	2 lymph nodes	*n (%)*	3 (9.1)	3 (9.1)	0 (0.0)	
	3 lymph nodes	*n (%)*	3 (9.1)	3 (9.1)	0 (0.0)	
**Excised total lymph node count** *(#)*		*Mean ± SD*	1.84 ± 0.99	2.21 ± 1.19	1.73 ± 0.90	^a^ 0.038 *
		*Median (min-max)*	2 (1–5)	2 (1–5)	2 (1–5)	
**Metastatic lymph node ratio** *(%)*		*Mean ± SD*	67.97 ± 28.55	67.97 ± 28.55	0 (0.0)	N/A
		*Median (min-max)*	60 (25–100)	60 (25–100)	0 (0.0)	
**Blue dye stained lymph node count** *(#)*		*Mean ± SD*	1.83 ± 0.99	2.21 ± 1.19	1.72 ± 0.91	^a^ 0.032 *
		*Median (min-max)*	2 (1–5)	2 (1–5)	1 (1–5)	
**Axillary lymph node dissection**	No	*n (%)*	149 (99.3)	32 (97.0)	117 (100.0)	^b^ 0.220
	Yes	*n (%)*	1 (0.7)	1 (3.0)	0 (0.0)	
**Surgical technique**	BCS & SLNB	*n (%)*	148 (98.7)	33 (100.0)	115 (98.3)	^b^ 1.000
	Oncoplastic surgery	*n (%)*	2 (1.3)	0 (0.0)	2 (1.7)	
**Mortality**	No	*n (%)*	149 (99.3)	33 (100.0)	116 (99.1)	N/A
	Yes	*n (%)*	1 (0.7)	0 (0.0)	1 (0.9)	
**Special** **pathological** **findings** *(n = 31)*	Multifocal	*n (%)*	2 (6.5)	0 (0.0)	2 (9.5)	^c^ 0.302
	Multicentric	*n (%)*	5 (16.1)	1 (10.0)	4 (19.0)	
	Solid papillary	*n (%)*	8 (25.8)	2 (20.0)	6 (28.6)	
	Micropapillary	*n (%)*	12 (38.7)	7 (70.0)	5 (23.8)	
	Encapsulated papillary	*n (%)*	2 (6.5)	0 (0.0)	2 (9.5)	
	Neuroendocrine	*n (%)*	2 (6.5)	0 (0.0)	2 (9.5)	

^a^ Mann–Whitney *U* test, ^b^ Fisher’s exact test, ^c^ Fisher–Freeman–Halton test, N/A: Not applicable, N: Total number of patients, n: number of patients, * *p* < 0.05, Mean ± SD: Mean ± standard deviation, Median (min-max): Median (minimum–maximum), BCS & SLNB: Breast conserving surgery and sentinel lymph node dissection.

**Table 3:** Diagnostic scan and receiver operating characteristic curve analysis for tumor size.

ROC Curve Analysis	Diagnostic Scan				ROC Curve		*p*
	Sensitivity *(%)*	Specificity *(%)*	PPV *(%)*	NPV *(%)*	Area	95% CI	
**Tumor size ≥ 19 mm**	57.58	59.83	28.79	83.33	0.603	0.520–0.682	0.070

ROC: Receiver operating characteristic, CI: Confidence interval, PPV: Positive predictive value, NPV: Negative predictive value.

**Table 4:** Multivariate analysis of the tumoral features that affect sentinel lymph node metastasis.

Tumoral Features		*p*	OR	95% CI for OR	
				*Lower*	*Upper*
**Histological grade**	Grade 1	0.366	Ref	Ref	Ref
	Grade 2	0.183	3.089	0.587	16.265
	Grade 3	0.163	3.557	0.599	21.126
**Tumor Size** *_(Millimeters)_*	≥19	0.368	1.484	0.629	3.503
**Ki-67** *(%)*	≥14	0.003 *	5.224	1.725	15.821

OR: Odds ratio, CI: Confidence interval, Ref: Reference, Ki-67: Antigen Kiel 67, * *p* < 0.01.

## Data Availability

The raw data supporting the conclusions of this article will be made available by the authors on request.

## Abbreviations

The following abbreviations are used in this manuscript:

LN	Lymph node
SLNB	Sentinel lymph node biopsy
BC	Breast cancer
BCS	Breast conserving surgery
PS	Permanent section pathological examination
ALND	Axillary lymph node dissection
FS	Frozen section pathological examination
IRB	Institutional review board
MG	Mammography
US	Ultrasonography
MRI	Magnetic resonance imaging
FNAB	Fine needle aspiration biopsy
RT	Radiotherapy
H&E	Hematoxylin–eosin
IHC	Immunohistochemical analysis
ITC	Isolated tumor cells
AJCC	American Joint Committee on Cancer
ER	Estrogen receptor
PR	Progesterone receptor
HER2	Human epidermal growth factor receptor 2
Ki-67	Antigen Kiel 67
NCSS	Number Cruncher Statistical System
PPV	Positive predictive value
NPV	Negative predictive value
ROC	Receiver operating characteristics
OR	Odds ratio
CI	Confidence interval
OS	Overall survival
LVI	Lymphovascular invasion
